# Strain-Dependent Responses of *Listeria monocytogenes* Biofilms to Combined Enzymatic and Bacteriocin Treatments

**DOI:** 10.3390/pathogens15101090

**Published:** 2026-10-17

**Authors:** Chin-Yi Chen, Joseph A. Capobianco, Joe Lee, Heather Koppenhöfer, Yiping He, Amanda Miller, Adam Oest, Kevin J. Lynn, Bryan W. Berger, John A. Renye

**Affiliations:** 1 Characterization and Interventions for Foodborne Pathogens Research Unit, Agricultural Research Service, United States Department of Agriculture, Wyndmoor, PA 19038, USA; 2 Dairy and Functional Foods Research Unit, Agricultural Research Service, United States Department of Agriculture, Wyndmoor, PA 19038, USA; 3 Department of Chemical Engineering, University of Virginia, Charlottesville, VA 22903, USA

**Keywords:** *Listeria monocytogenes*, biofilm, bacteriocin, anti-biofilm treatments

## Abstract

*Listeria monocytogenes* forms resilient biofilms that protect cells from sanitizers and environmental stress, allowing the pathogen to persist in food processing environments. This study evaluated the combined effects of an engineered carbohydrate-active enzyme (CAase) and two food-grade bacteriocins, nisin and thermophilin 110 (T110), on biofilm disruption and cell inactivation. Biofilm biomass was quantified by crystal violet staining and visualized by microscopic imaging, with viable cells enumerated in treatment supernatants. Minimum inhibitory concentrations (MIC) for nisin (12.5–25 µg mL^−1^) and T110 (64 to 512 arbitrary units (AU) mL^−1^) were determined against all 11 *L. monocytogenes* strains, while CAase alone showed limited antimicrobial activity. CAase was shown to significantly reduce biofilm biomass, releasing embedded cells from the matrix. Nisin (25 µg mL^−1^) and T110 (1024 AU mL^−1^) were observed to decrease biofilm biomass and viable-cell recovery, but results were strain-dependent. Biofilm clearance improved for some *L. monocytogenes* strains when CAase was combined with either bacteriocin, but nisin was more effective at reducing the number of viable cells recovered. Results from this study demonstrated that enzymatic degradation of the extracellular matrix may enhance bacteriocin efficacy by improving access to target cells and support the potential of enzyme–bacteriocin combinations as food-safe biofilm control strategies in food production environments.

## 1. Introduction

*Listeria monocytogenes* (*L. monocytogenes*) is a ubiquitous bacterium found within soil, plants, water, and animal feces, and can enter food processing facilities in a variety of ways, including via raw materials and their contact with contaminated surfaces [1]. *L. monocytogenes* infections are rare, with pregnant women, newborns, and elderly or immunocompromised individuals being most susceptible. Infection typically occurs through the ingestion of contaminated foods, particularly raw or inadequately processed products, and can lead to listeriosis, a life-threatening infection [2] with approximately 1250 cases and 172 deaths each year [3]. Although *L. monocytogenes* is considered sensitive to thermal food processing procedures, including pasteurization, adaptive heat responses have been reported [4], and its psychrotolerant and halotolerant phenotypes allow it to persist within foods and grow at refrigeration temperatures [5]. Additionally, *L. monocytogenes* has been reported to form persistent biofilms on surfaces commonly found in food manufacturing environments [6,7], allowing for cross-contamination of processed foods. These biofilms can develop on stainless steel, rubber, and plastic components within processing lines and are often located in hard-to-clean areas such as gaskets, drains, and equipment seams [8,9]. Within a biofilm, bacterial cells are enclosed in an extracellular polymeric substance (EPS) that shields them from sanitizers, mechanical cleaning, and environmental stress [10,11]. As a result, *L. monocytogenes* can survive standard sanitation cycles, recolonize processing equipment, and cause recurrent contamination events [8,12].

Traditional cleaning and disinfection programs rely on detergent washing followed by application of chemical sanitizers such as chlorine, peracetic acid, or quaternary ammonium compounds [9,13]. While these agents are generally effective against planktonic cells, their ability to penetrate mature biofilms is limited. Cells embedded within the EPS may persist in a dormant or metabolically altered state, allowing them to survive treatment and later re-establish contamination once favorable conditions return [10,11,13]. These limitations have created growing interest in control strategies that directly target the biofilm matrix, removing or inactivating protected cells without damaging equipment or leaving chemical residues [13].

Enzymatic treatments offer a promising alternative for biofilm control because they act specifically on the structural components that stabilize the matrix [13,14]. Enzymes that hydrolyze carbohydrate linkages can weaken the matrix and promote detachment of embedded cells [13,14]. Previous work demonstrated that an engineered carbohydrate-active enzyme (CAase) was able to degrade the *L. monocytogenes* biofilm matrix [15]. That study showed that CAase treatment reduced biofilm biomass and enhanced the antimicrobial activity of the class IIa bacteriocin thermophilin 110 (T110) against strain ATCC 19111. In related work, CAase increased recovery of *L. monocytogenes* from biofilm samples used for detection, further confirming its ability to act on the matrix [16]. Together, these findings established that CAase can weaken *L. monocytogenes* biofilms, though previous studies were limited to a single strain and did not assess broader applicability across diverse isolates.

Bacteriocins such as nisin and T110 are small antimicrobial peptides produced by lactic acid bacteria and are considered safe for use in foods [17,18]. Nisin, a class I bacteriocin, disrupts cell membranes through pore formation and interrupts peptidoglycan production [19], while T110 is an example of a class IIa bacteriocin, which is similar to pediocin [18]. Nisin has been shown to utilize lipid II as a docking molecule on susceptible bacteria, while class II bacteriocins, including pediocin, have been shown to target the mannose phosphotransferase receptor, resulting in membrane depolarization and cell death [17,18,19,20]. Although T110 is a class IIa bacteriocin, the docking molecule required for its antimicrobial activity has not been confirmed. While both peptides show strong activity against planktonic *L. monocytogenes* [17,21], reports have suggested they are less effective alone against cells within a mature biofilm [15,22]. Combining a biofilm-degrading enzyme with a bacteriocin, therefore, provides a logical strategy to enhance antimicrobial performance by breaking down the matrix and potentially providing more access to cells [13,15].

The present study builds on the previous findings that CAase and T110 were effective at disrupting *L. monocytogenes* 19111 biofilms [15]. The current study evaluates the effects of CAase, nisin, and T110, individually, and the combinations of CAase and each bacteriocin against a diverse panel of *L. monocytogenes* strains. Biofilm disruption was quantified by crystal violet staining and visualized by microscopy imaging to assess treatment effects on biofilm structure. The viability of cells released from treated biofilms was determined by plating supernatants, and the antimicrobial activity of each agent against planktonic cells was assessed using minimum inhibitory concentration (MIC) and minimum bactericidal concentration (MBC) assays. By including multiple strains representing different lineages and environmental origins, this work examined whether the complementary enzyme + bacteriocin mechanism previously demonstrated for strain 19111 extends to a broader range of isolates. The findings provide insight into strain-dependent variability in biofilm treatment response and demonstrate the potential of enzyme–bacteriocin combinations as food-safe tools for biofilm control in food production environments.

## 2. Materials and Methods

### 2.1. Reagents and General Procedures

Nanopure water was prepared using a Barnstead ultrapure system (Thermo Fisher Scientific, Canoga Park, CA, USA). Phosphate-buffered saline (PBS) was prepared from commercial tablets (Sigma-Aldrich, St. Louis, MO, USA) and sterilized by 0.2 µm filtration (Corning, Glendale, AZ, USA). Luria–Bertani (LB) broth and LB agar (Becton Dickinson, Franklin Lakes, NJ, USA) were prepared according to manufacturer instructions. TYL broth (tryptone–yeast–lactose) was used for cultivation of *Streptococcus thermophilus*. All culture media were sterilized by autoclaving. Crystal violet (Millipore Sigma, Burlington, MA, USA) was prepared fresh at 0.1% (*w*/*v*) for biofilm staining.

### 2.2. Bacterial Strains and Cultivation

Eleven *L. monocytogenes* strains were examined: 10403S, EGDe, F2365, 19111, 19113, 19117, 19118, BAA-3131, BAA-3132, BAA-3134, and BAA-3135 (Table 1). Cultures were grown aerobically overnight at 37 °C in LB broth. *S. thermophilus* strain B59671 (T110 producer) was maintained in tryptone-yeast extract-lactose (TYL) broth at 37 °C. Prior to use, all bacterial strains were stored at −80 °C in their respective growth medium supplemented with 15% glycerol.

### 2.3. Preparation of Enzyme and Bacteriocins

CAase was produced by recombinant expression in *E. coli* BL21(DE3) carrying a codon-optimized gene (pET-28a(+)), induced with IPTG, and purified by Ni-NTA affinity chromatography. Purity was monitored by SDS–PAGE and protein quantified spectrophotometrically [14]. Nisin (Sigma-Aldrich, St. Louis, MO, USA) stocks were prepared at 400 µg mL^−1^ in DMSO, clarified by centrifugation (12,000× *g*, 5 min), stored at −20 °C, and diluted in sterile PBS immediately before use. Thermophilin 110 (T110) was partially purified from cell-free supernatant (CFS) collected after *S. thermophilus* B59671 was grown in TYL overnight at 37 °C. Bacteriocin was recovered using a previously described chloroform extraction protocol [18]. The antimicrobial activity of the T110 preparation was determined by preparing serial two-fold dilutions of the bacteriocin preparation and using 50 µL of each dilution in a well diffusion assay with *S. thermophilus* ST113 as the target bacterium. Arbitrary units (AU) mL^−1^ were calculated as the reciprocal of the highest dilution multiplied by 20. A stock of T110 was prepared at 5120 AU mL^−1^ and stored at −20 °C.

### 2.4. Minimum Inhibitory Concentration (Mic) and Minimum Bactericidal Concentration (Mbc) Assays

Two-fold serial dilutions of CAase (1–0.0156 mg mL^−1^), nisin (200–3.125 µg mL^−1^), or T110 (2048–31.25 AU mL^−1^; 2.5- to 160-fold dilution of a 5120 AU mL^−1^ stock) were prepared in LB broth in 96-well microplates. Wells were inoculated with ~2 × 10^6^ CFU mL^−1^ of *L. monocytogenes* and incubated aerobically at 30 °C for up to 24 h. Growth inhibition was assessed visually and by measuring optical density (OD_600_) using a Cytation 5 multimode plate reader (Agilent Bioteck, Santa Clara, CA, USA). The MIC was defined as the lowest concentration without detectable growth. To determine MBC, 10 µL from wells at and around the MIC (one below and two to three above) were plated on LB agar and incubated at 30 °C for 24 h; the MBC was the lowest concentration yielding no colonies. All MIC/MBC assays were performed in triplicate.

### 2.5. Biofilm Formation and Treatment

Cell densities for the 11 overnight *L. monocytogenes* cultures were determined to be ~1 × 10^9^ CFU mL^−1^. Cultures were diluted 500-fold, resulting in an initial inoculum of ~2 × 10^6^ CFU mL^−1^ in LB. Aliquots (100 µL) were dispensed into sterile, flat-bottom 96-well polystyrene plates (12 wells per strain) and incubated at 30 °C for six days in a sealed, humidified chamber without disturbance, as previously described [15]. After biofilm maturation, wells were gently aspirated to remove planktonic cells, then treated in triplicate with PBS (control), CAase (0.1 mg mL^−1^), T110 (1024 AU mL^−1^), nisin (25 µg mL^−1^), or CAase combined with either bacteriocin. Treatments were incubated statically for 20–24 h at 30 °C.

### 2.6. Crystal Violet Biofilm Biomass Assay

Following treatment, supernatants were removed for viability testing. Biofilms were washed three times with sterile PBS and stained with 0.1% crystal violet (150 µL per well) for 30 min at room temperature. Excess crystal violet was removed by rinsing the wells three times with distilled water, and then air-dried for ~15 min. Whole-well images of stained biofilms were captured with an oCelloScope (BioSense Solutions, Farum, Denmark) using UniExplorer software (v. 12.0) in Full-well Acquire mode. After imaging, bound dye was solubilized with 100% ethanol (150 µL per well, 30 min), and absorbance at 590 nm was measured on a Cytation 5 multimode plate reader (Agilent/Biotek, Santa Clara, CA, USA) using Gen5 software (ver. 3.12.08).

### 2.7. Enumeration of Detached and Surviving Cells

Supernatants from treated wells were collected, serially diluted ten-fold in LB (10^−1^ to 10^−6^), and 10 µL drops from each dilution were plated (six drops per dilution) onto BHI agar. Plates were incubated overnight at 30 °C, and colonies were counted to determine viable CFU. Counts were normalized to PBS controls for each strain to obtain relative recovery.

### 2.8. Statistical Analysis

All quantitative data were analyzed using JMP (v19.0.4, SAS Institute Inc., Cary, NC, USA). For each strain and treatment, means and standard deviations were calculated from at least three independent replicates. Prior to comparison, data were evaluated to confirm that assumptions for parametric testing were satisfied. Normality was assessed using the Shapiro–Wilk test, and equality of variances was evaluated using Levene’s test, as implemented in JMP (SAS Institute, Cary, NC, USA). When these assumptions were met, two-tailed Student’s *t*-tests were used to compare each treatment with its corresponding PBS control. Statistical significance was defined as *p* < 0.05. Graphical summaries were generated in JMP, and connecting letters indicating significant differences were added manually to exported figures for clarity of presentation.

## 3. Results

### 3.1. Minimum Inhibitory Concentration (Mic) of Nisin, T110 and CAase Against L. monocytogenes

The inhibitory activity of nisin, T110, and CAase against planktonic *L. monocytogenes* was evaluated by measuring optical density (OD_600_) after incubation with serial two-fold dilutions of each agent. MIC determination was made based on visual inspection of culture growth and OD_600_ measurements (Table 2). Nisin produced the most consistent inhibitory profile among the two bacteriocins tested. Growth of all *L. monocytogenes* strains declined sharply between 6.25 and 25 µg mL^−1^, after which OD values stabilized, indicating complete inhibition. While most strains exhibited a MIC of 12.5 µg mL^−1^, strains BAA-3131, BAA-3132, and BAA-3134 required a nisin concentration of 25 µg mL^−1^ for growth inhibition. Unlike nisin, the concentration of T110 required to inhibit growth varied significantly for *L. monocytogenes* strains, with some exhibiting intermediate levels of inhibition based on OD_600_ measurements (e.g., 19113, BAA-3132, and EGDe). T110 was shown to inhibit the growth of most *L. monocytogenes* strains at ≤256 AU mL^−1^, with strains 19113 and F2365 being exceptions, requiring a T110 concentration ≥ 512 AU mL^−1^ to completely inhibit growth (Table 2). Strain 19111 was the most sensitive to T110, with a MIC of 64 AU mL^−1^.

CAase was also assessed for its ability to inhibit *L. monocytogenes* growth. Although planktonic cultures for all strains appeared less dense when treated with ≥0.5 mg mL^−1^ CAase, complete growth inhibition was not observed for any strain at the highest concentration (1 mg mL^−1^) tested. These results indicate that CAase is more valuable as an enzyme that disrupts biofilms at significantly lower concentrations (0.1 mg mL^−1^).

Based on the above results, it was decided that biofilm assays would use 0.1 mg mL^−1^ of CAase, which was previously reported to disrupt *L. monocytogenes* biofilms [15]; and nisin would be used at 25 µg mL^−1^, the highest MIC recorded in this study. Due to the efficacy of T110 against planktonic *L. monocytogenes* cells being more variable when compared to nisin, a concentration 2-fold above the highest MIC observed (1024 AU mL^−1^) was selected for use in biofilm studies.

### 3.2. Biofilm Disruption Measured by Crystal Violet Absorbance

Crystal violet staining was used to quantify residual biofilm biomass following treatment with CAase, nisin, or T110 alone, and the combination of CAase with either nisin or T110. Biofilms were established from eleven *L. monocytogenes* strains and exposed to each treatment for 20–24 h before staining. Absorbance at 590 nm was used to estimate total biofilm biomass, and results were analyzed by Student’s *t*-tests (α = 0.05) to identify significant differences within each strain.

**Figure 1:**
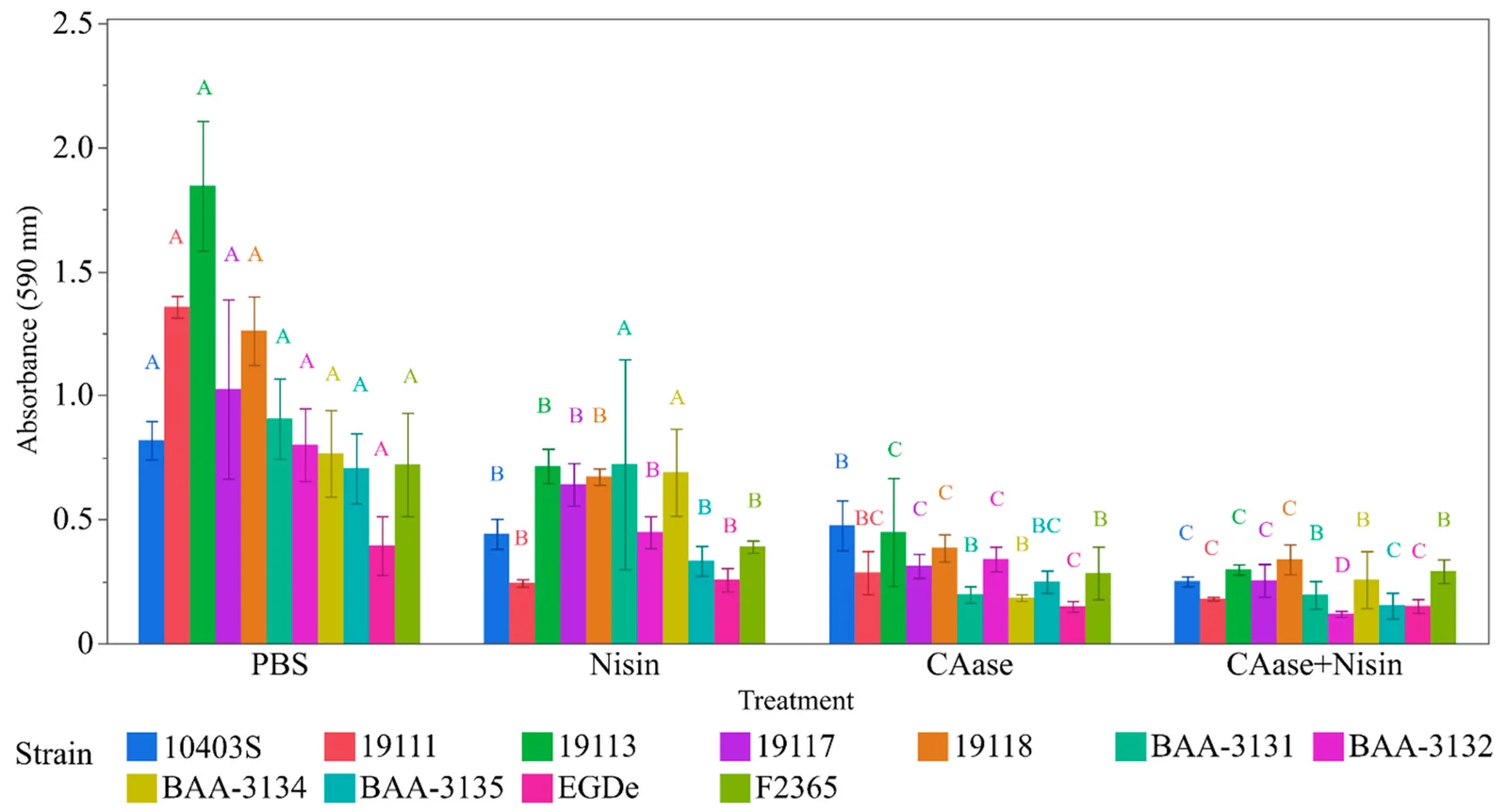
Quantification of *Listeria monocytogenes* biofilm biomass following treatment with CAase, nisin, and their combination. Biofilms of eleven *L. monocytogenes* strains were treated with phosphate-buffered saline (PBS; control), nisin (25 µg mL^−1^), CAase (0.1 mg mL^−1^), or CAase plus nisin for 20–24 h at 30 °C. Residual biofilm biomass after treatment was quantified by crystal violet staining and measurement of absorbance at 590 nm. Bars represent mean ± standard deviation (n ≥ 6, depending on treatment). Within each strain, treatments not sharing the same letter differ significantly (*p* < 0.05, Student’s *t*-test).

Nisin was investigated for its potential to assist CAase in the disruption of *L. monocytogenes* biofilms since it is commercially available for use as a food-grade antimicrobial (Figure 1). Baseline biofilm formation varied among strains, with absorbance values for the PBS controls ranging from 0.39 ± 0.12 for EGDe to 1.84 ± 0.26 for strain 19113. Treatment with CAase alone significantly reduced biofilm biomass in all strains (*p* < 0.05), indicating enzymatic degradation of the biofilm matrix. The extent of reduction varied between isolates; for example, strain 10403S decreased from 0.82 ± 0.08 in PBS to 0.48 ± 0.10, while strains 19111 and 19113 showed more pronounced decreases from 1.36 ± 0.04 in PBS to 0.29 ± 0.09 and 1.84 ± 0.26 in PBS to 0.45 ± 0.22 after CAase treatment (*p* < 0.05). Treatment with nisin alone led to a significant reduction in biofilm biomass for nine of the strains tested. Biomass for the remaining two strains, BAA-3131 and BAA-3134, was comparable to the PBS control. When CAase and nisin were applied together, residual biomass dropped to its lowest value for two strains, 10403S and BAA-3132. For the remaining nine strains, the inclusion of nisin did not result in a significant drop in biomass from what was observed using CAase alone. These results suggested that the combined use of CAase and nisin was more effective than either treatment alone for ensuring an optimal reduction in most *L. monocytogenes* biofilms.

**Figure 2:**
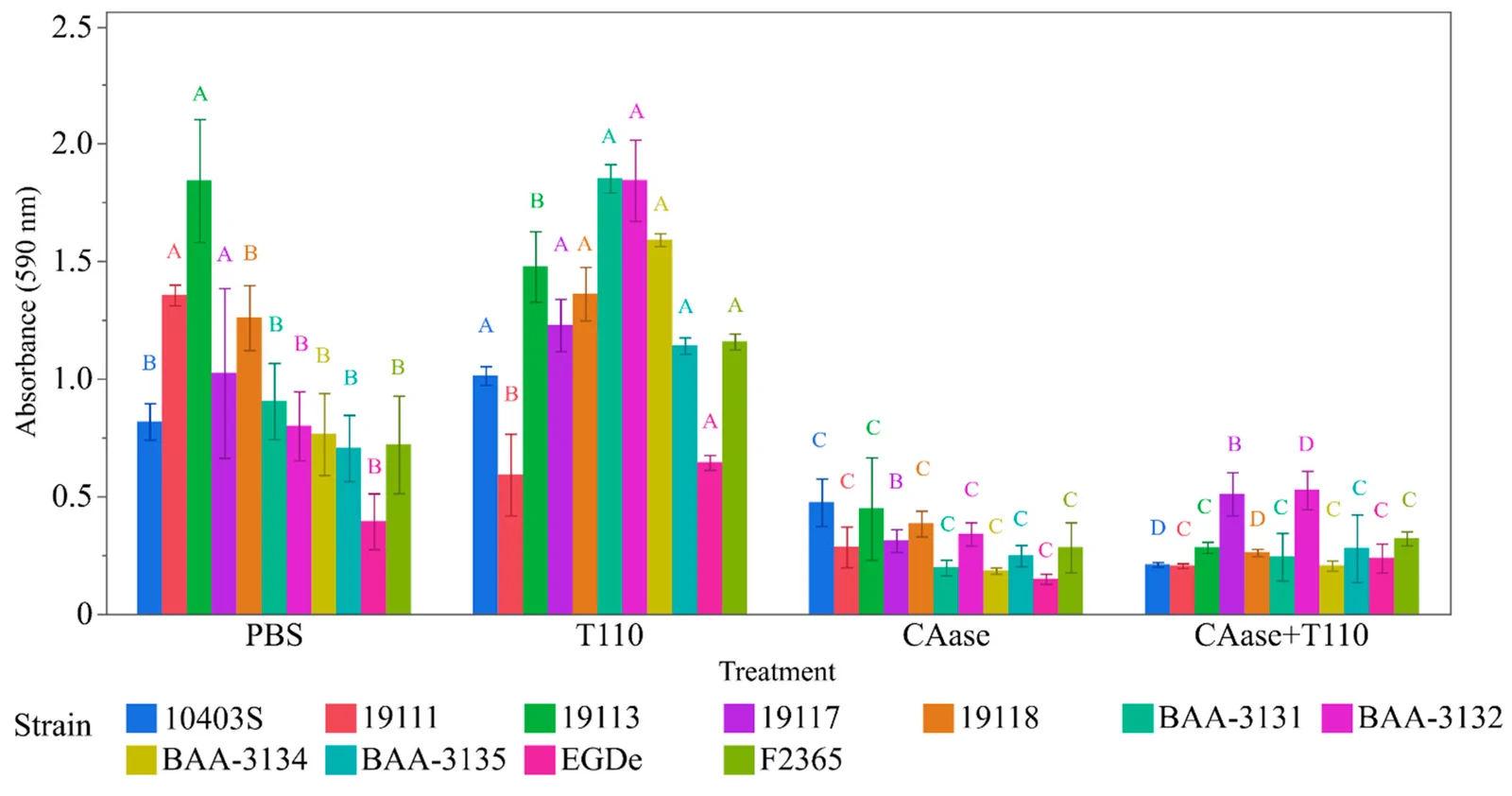
Quantification of *Listeria monocytogenes* biofilm biomass following treatment with CAase, T110, and their combination. Biofilms of eleven *L. monocytogenes* strains were cultivated under standard conditions and treated with phosphate-buffered saline (PBS; control), T110 (1024 AU mL^−1^), CAase (0.1 mg mL^−1^), or a combination of CAase and T110 for 20–24 h at 30 °C. Residual biofilm biomass after treatment was quantified by crystal violet staining and measurement of absorbance at 590 nm. Bars represent mean ± standard deviation (n ≥ 3, depending on treatment). Within each strain, treatments not sharing the same letter differ significantly (*p* < 0.05, Student’s *t*-test).

T110 was also assessed for the potential to disrupt mature *L. monocytogenes* biofilms (Figure 2). When used alone, T110 was shown to be significantly less effective at biofilm disruption than CAase (Figure 2) or nisin (Figure 1) alone. Just two strains, 19111 (0.59 ± 0.17) and 19113 (1.48 ± 0.15), exhibited statistically significant decreases in biomass when compared with their PBS controls. Eight of the remaining strains displayed increased biomass following exposure to T110 alone, with BAA-3131 (1.85 ± 0.06) and BAA-3132 (1.85 ± 0.17) showing the highest absorbance values. When CAase and T110 were applied together, strains 10403S and 19118 showed their lowest residual biomass values, suggesting the use of both CAase and T110 could improve the efficacy of biofilm disruption for some strains. For most strains, the addition of T110 did not enhance biofilm disruption significantly when compared to CAase treatment alone. For strains 19117 and BAA-3132, the inclusion of T110 appeared to exert the opposite effect, with slightly higher biomasses observed. Although the T110 concentration used for biofilm disruption was two-fold higher than the determined MIC value, results from these assays suggest that higher concentrations may be needed for activity against biofilm-encapsulated cells.

**Figure 3:**
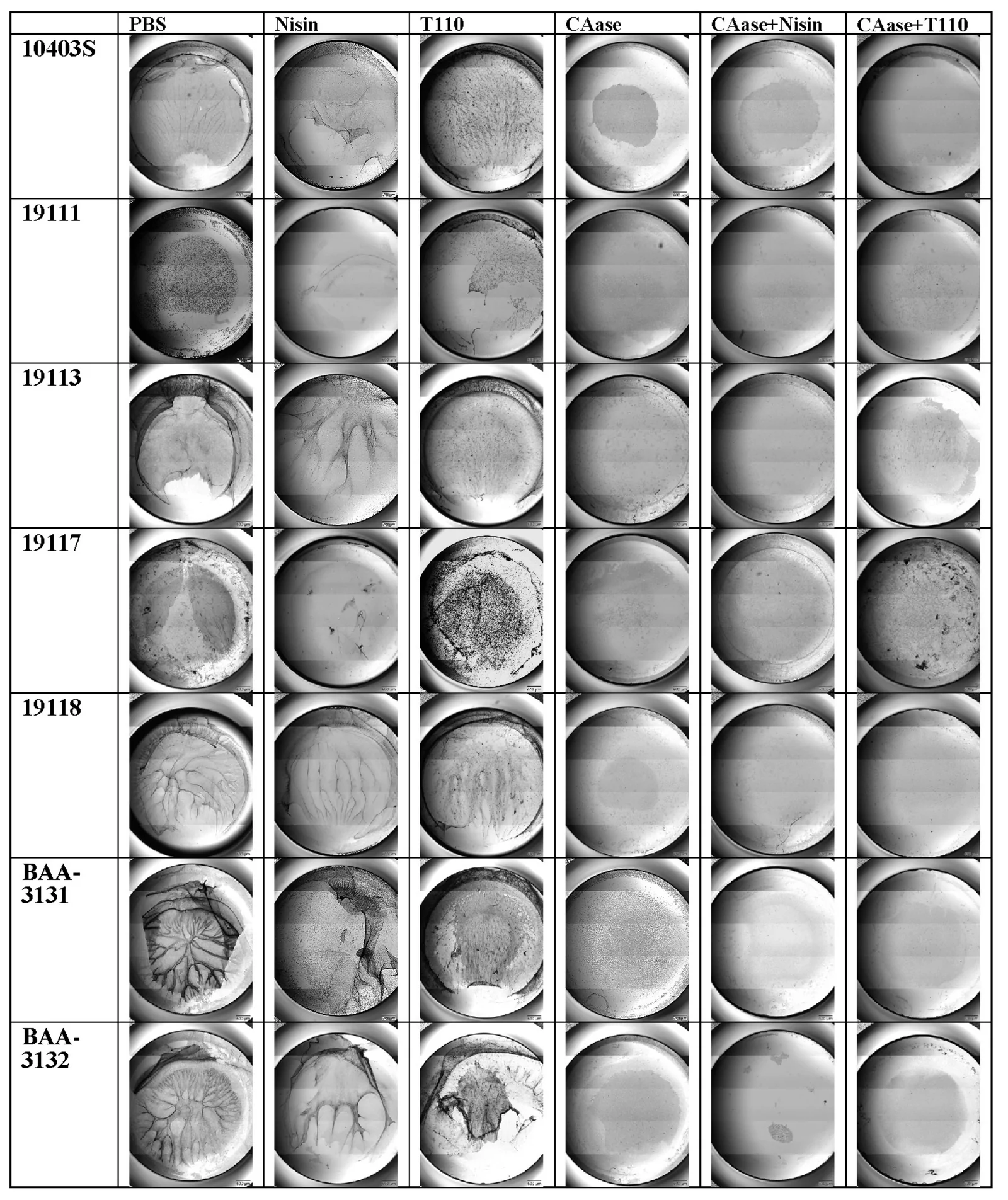

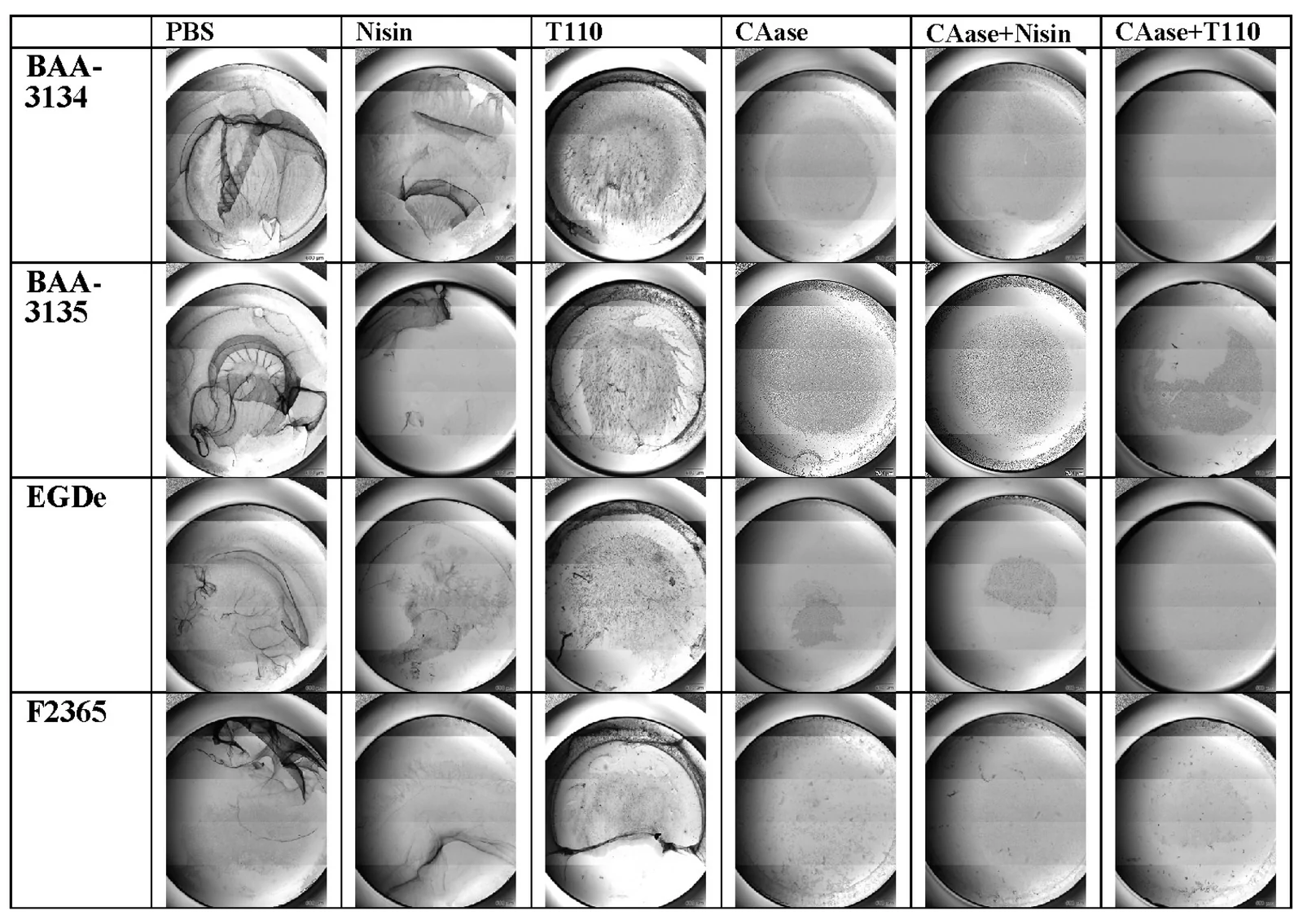
Images of crystal violet-stained *Listeria monocytogenes* biofilms following treatment with CAase and bacteriocins. Representative images show *L. monocytogenes* biofilms exposed to PBS (control), nisin, T110, CAase, or combinations of CAase with nisin or T110. The panel to left of the well images lists the *Listeria monocytogenes* strains: 10403S, 19111, 19113, 19117, 19118, BAA-3131, BAA-3132, BAA-3134, BAA-3135, EGDe, and F2365. Each image represents the full interior of a 96-well plate well. Scale bar for all images is 600 µm.

To visually assess the effect of CAase and bacteriocins against mature biofilms, crystal violet-stained *L. monocytogenes* biofilms were imaged following treatments. In general, these images agreed with the quantitative measurements of biofilm biomass presented in Figure 1 and Figure 2. In control wells (PBS), dense, mesh-like biofilm layers were observed, although biomass and community structure appeared to differ between strains (Figure 3). For example, 19117 and BAA-3131 both formed dense biofilms, but their overall structure appeared to differ when observed microscopically. When biofilms were exposed to bacteriocins alone, reductions in biomass could be observed, but results varied depending on the bacteriocin used and the target strain. For example, CV absorbance readings showed that biofilms formed by strains 19111 and 19113 had significantly reduced biomass when exposed to either nisin (Figure 1) or T110 (Figure 2), which correlates with the images in Figure 3. Although the biofilm appeared less dense when treated with T110 alone, the residual biomass still appeared higher when compared to nisin treatment alone. For strain 19117, microscopic imaging clearly shows that nisin was more effective at reducing the attached biofilm when compared to T110 alone, which also agreed with the quantitative results reported in Figure 1 and Figure 2. The most dramatic differences in biofilm imaging were observed when CAase was applied alone or in combination with nisin or T110. For most strains, residual biomass was minimal, and the loss of mesh-like materials was pronounced when CAase treatment was applied, with most of the remaining stained biomass concentrated in the center of the well. Additionally, when CAase and T110 were used together, a higher amount of residual biomass remained visible in the well when compared to CAase treatment alone. This was most noticeable for strain 19117, again agreeing with the quantitative results reported in Figure 2. Collectively, these results demonstrated that CAase weakens and disrupts *L. monocytogenes* biofilms, and that bacteriocin co-treatment could enhance the reduction of these biofilms depending on the strain targeted.

### 3.3. Viable L. monocytogenes Cells Recovered in Supernatant After Treatment

**Figure 4:**
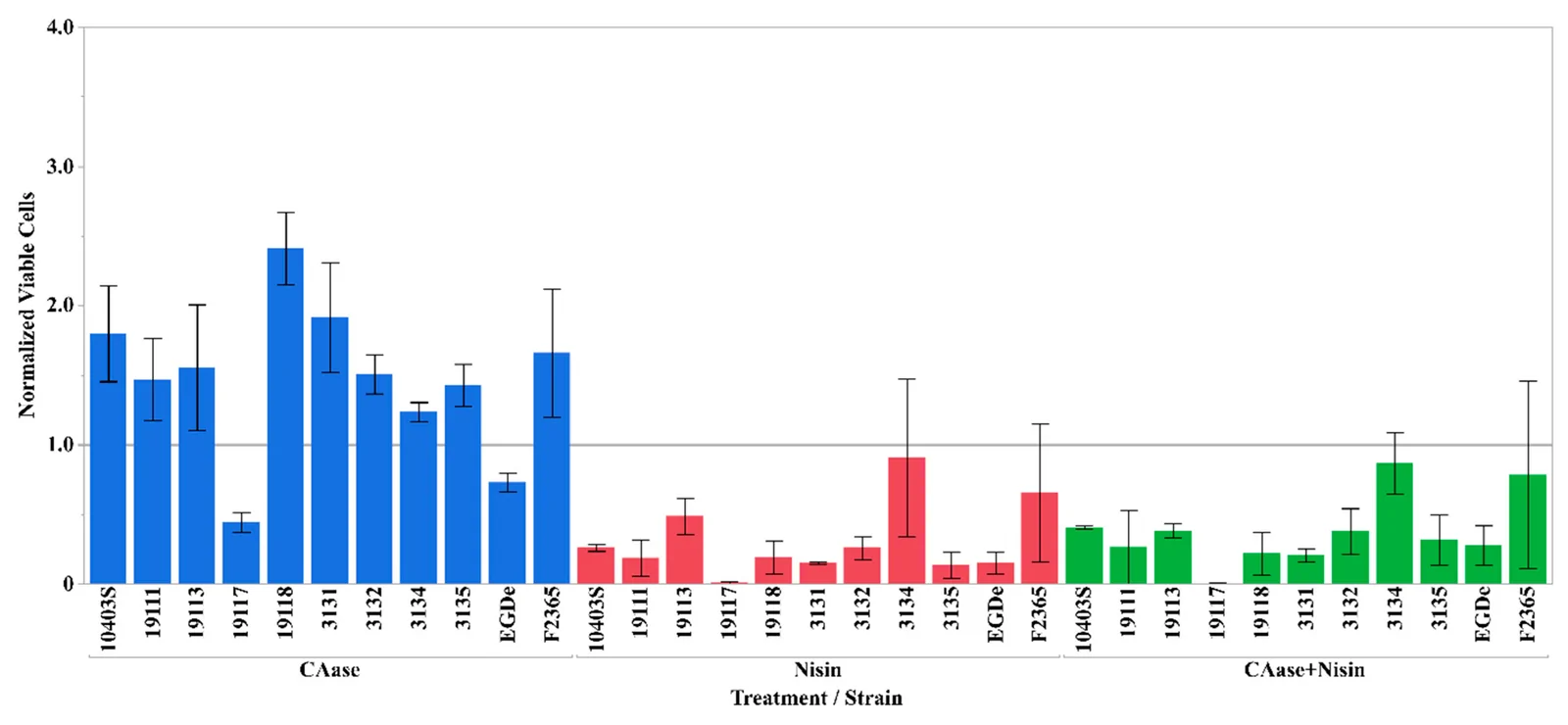
Normalized recovery of viable *Listeria monocytogenes* cells from biofilm supernatants following treatment with CAase, nisin, or their combination. Biofilms were treated with CAase (0.1 mg mL^−1^), nisin (25 µg mL^−1^), or CAase plus nisin for 20–24 h at 30 °C. Supernatants were plated for colony enumeration, and CFU counts were normalized to those from PBS controls. Bars represent mean ratios of treatment CFU to PBS control CFU for individual strains. Error bars represent 1 standard error. The horizontal line at 1.0 indicates equivalence with the control. Treatments are shown with different color bars: CAase alone (blue), nisin alone (red) and CAase+nisin (green).

**Figure 5:**
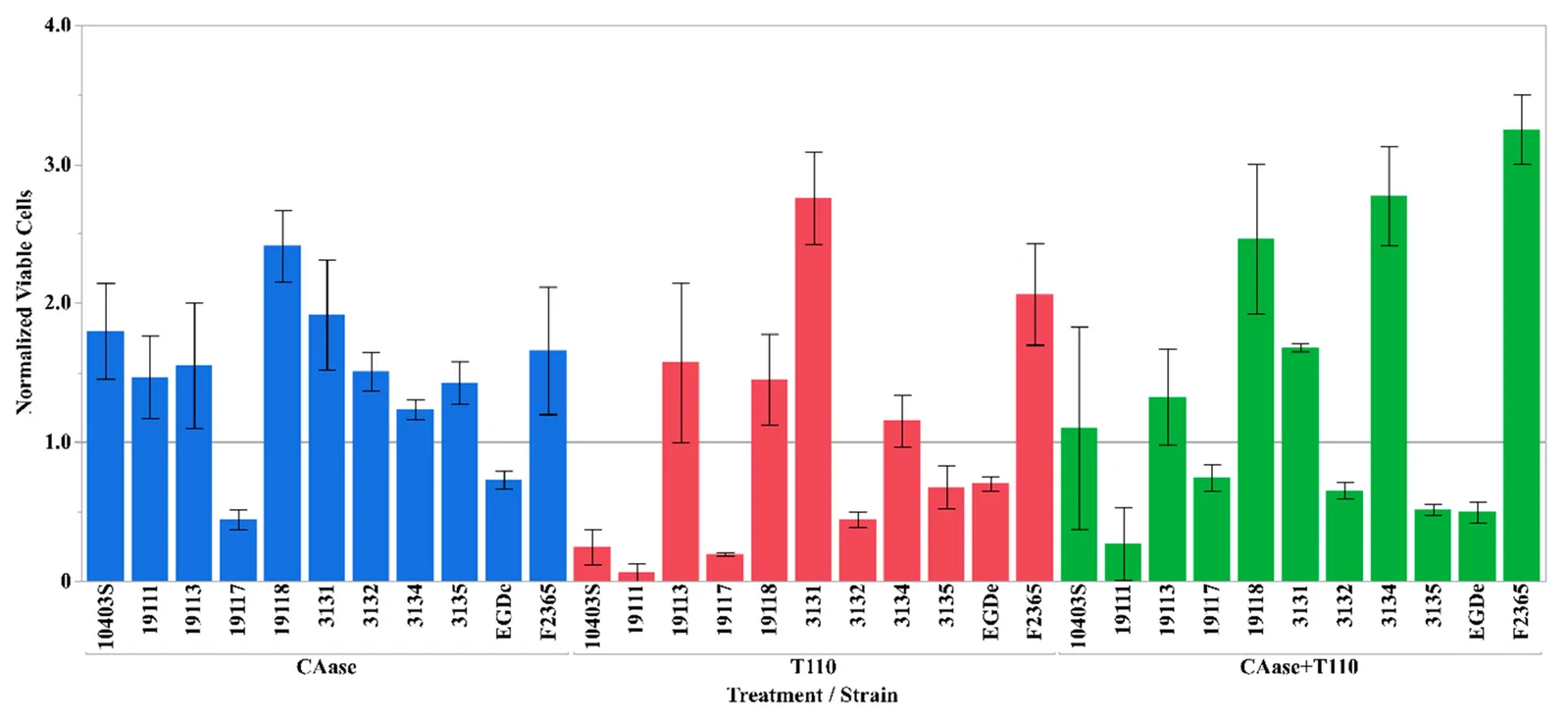
Normalized recovery of viable *Listeria monocytogenes* cells from biofilm supernatants following treatment with CAase, T110, or their combination. Biofilms were treated with CAase (0.1 mg mL^−1^), T110 (1024 AU mL^−1^), or CAase plus T110 for 20–24 h at 30 °C. Supernatants were plated for colony enumeration, and CFU counts were normalized to those from PBS controls. Bars represent mean ratios of treatment CFU to PBS control CFU for individual strains. Error bars represent 1 standard error. The horizontal line at 1.0 indicates equivalence with the control. Treatments are shown with different color bars: CAase alone (blue), nisin alone (red) and CAase+nisin (green).

To determine if the assessed treatments resulted in the dispersion of viable *L. monocytogenes* cells, supernatants from treated biofilms were collected and plated for colony enumeration. Colony counts were normalized to those from the corresponding PBS control for each strain. Results are expressed as the ratio of CFU mL^−1^ in the treated wells to the control (PBS) wells. A ratio of 1.0 indicates equivalence with the PBS control, representing the number of viable cells that are naturally shed from a mature biofilm (horizontal line in Figure 4 and Figure 5).

For most strains, treatment with CAase alone produced ratios greater than 1, indicating the release of viable cells from biofilms via the enzymatic degradation of the biofilm matrix. The magnitude of this increase varied among strains, and surprisingly, strains 19117 and EGDe showed fewer viable cells released after exposure to CAase when compared to the PBS control. This could suggest that the biofilm matrices for 19117 and EGDe are less susceptible to enzymatic degradation by CAase, but this does not agree with CV staining and microscopy results (Figure 1, Figure 2 and Figure 3), which suggested CAase effectively reduced the biofilm biomass for both strains. However, since CV stains both cells and EPS, the discrepancy could be due to the complexity of their EPS matrix. Loss of EPS would factor into the results observed in Figure 1, Figure 2 and Figure 3 but may not accurately represent the number of cells within the intact biofilm. It’s possible that cells are adhered tightly to the bottom of the well and not released with the degradation of EPS. Viability counts could be used to determine the number of viable cells remaining within the residual biomass, but that was not done in the current study because our goal was to assess both the amount and structure of the residual biomass after treatment and to correlate those results with the number of viable cells released from the same biofilm. Additional studies are required to determine if 19117 or EGDe cells adhere more strongly to surfaces than the other *L. monocytogenes* strains used in this study.

Treatment with nisin alone resulted in a lower number of viable cells being recovered in supernatants when compared to the untreated control for all strains tested (Figure 4, horizontal line). However, Figure 1 showed that nisin alone significantly reduced the biomass of *L. monocytogenes* biofilms, suggesting the concentration of nisin used in this study was sufficient to inhibit the growth of bacteria released from mature biofilms or kill cells within the biofilm, leading to the shedding of dead cells. A similar result was observed when nisin was combined with CAase. For all strains tested, the combined treatment did not result in the release of more viable cells than the PBS control, suggesting nisin inhibited the growth of dispersed cells. Strains BAA-3134 and F2365 showed the highest number of viable cells released after treatment with nisin alone or in combination with CAase, suggesting a higher concentration of nisin may be needed to inhibit their growth.

When biofilms were treated with T110 alone, viable cell numbers within supernatants were similar to or higher than the control (PBS) treatment for five *L. monocytogenes* strains, 19113, 19118, BAA-3131, BAA-3134, and F2356 (Figure 5). Although T110 did not significantly reduce biofilm biomass for these strains based on CV staining (Figure 2), results suggest that T110 may partially disrupt the biofilm, leading to the release of viable cells. These results also suggest that the concentration of T110 used for biofilm treatment assays (1024 AU mL^−1^) was not sufficient for inhibiting the growth of the released cells, especially for these five strains which had high MICs (Table 2). Although the concentration of T110 was at least 2-fold higher than the MIC values for each of these strains, it is possible that their growth within biofilms altered their susceptibility to T110. For the remaining six strains, viable cells within supernatants were lower than that observed in the control (PBS), suggesting T110 inhibited the growth of planktonic cells released from the biofilm.

Similar results were observed when T110 was combined with CAase for biofilm dispersion, with six strains (10403S, 19113, 19118, BAA-3131, BAA-3134 and F2356) showing similar or more viable cells within supernatants when compared with the PBS-treated biofilms. For some strains, such as BAA-3134 and F2356, the number of viable cells released using the combined treatment was higher than the number observed when either T110 or CAase was assayed alone (Figure 5). This suggests the antimicrobial activity of T110 was not effective in killing the elevated number of cells released by CAase treatment. For other strains, such as BAA-3131, the number of viable cells recovered from supernatants after the combined treatment was lower when compared to a biofilm treated with T110 alone, indicating T110 inhibited the growth of CAase-released BAA-3131 cells. Viable cell counts within supernatants for the remaining strains (19111, 19117, BAA-3132, BAA-3135 and EGDe) after the combined CAase and T110 treatment remained lower than the control, suggesting T110 was able to inhibit the growth of cells released from these biofilms.

The number of viable cells observed within supernatants following T110 treatments was surprising since the concentration used in biofilm assays was at least two-fold higher than the MIC values determined in Table 2. To investigate this further, the minimum bactericidal concentration (MBC) for T110 against all *L. monocytogenes* strains was determined. The MBC differed significantly from the MIC values determined in Table 2. For many *L. monocytogenes* strains, the MBC was determined to be ≥2048 AU mL^−1^, which was two-fold higher than the concentration of T110 used in the study. This suggests a higher concentration of T110 may be required to kill *L. monocytogenes* cells released when CAase is used to disperse biofilm cells.

When the MBC was determined for nisin against the 11 *L. monocytogenes* strains used, the value was determined to be ≤25 µg mL^−1^. The use of 25 µg mL^−1^ of nisin within the biofilm assays is close to the MBC for all strains, thus making nisin a more effective antilisterial under the conditions used in this study.

## 4. Discussion

Results of this study demonstrated the potential for bacteriocins, specifically nisin and T110, to enhance CAase-induced dispersion of *Listeria monocytogenes* biofilms. CAase alone consistently weakened or disrupted the biofilm matrix for all *L. monocytogenes* strains tested in this study, producing measurable reductions in crystal-violet staining and visible thinning of surface-associated material. These results agree with previous reports showing that CAase could degrade the EPS component of mature *Escherichia coli*, *Salmonella* Typhimurium, *Pseudomonas fluorescens,* and *L. monocytogenes* biofilms [14,15,25]. While CAase-induced biofilm dispersion has been shown to have practical application for enhancing the detection of *L. monocytogenes* in food products [16], its use within intervention technologies requires further investigation. Since CAase has little or no antimicrobial activity alone, additional antimicrobials can be combined with CAase to kill bacterial cells released from within biofilms. A previous study showed that CAase in combination with peracetic acid (PAA) was effective at reducing the number of viable *S*. Typhimurium and *P. fluorescens* within biofilms on commonly used food manufacturing surfaces, including stainless steel, silicone, and nylon [25]. However, there are safety concerns with the use of PAA; thus, the potential for food-grade antimicrobials to enhance CAase activity was explored. Previously, it was shown that the combined use of CAase and a *S. thermophilus* bacteriocin, T110, enhanced biofilm removal by improving antimicrobial access to cells previously protected within the extracellular matrix [15]. However, this study only assessed activity against *L. monocytogenes* strain 19111 and did not evaluate the viability of cells released from the mature biofilm. The current study sought to determine the efficacy of using a combination of CAase and food-grade bacteriocins, T110 and nisin, to disperse mature *L. monocytogenes* biofilms and inhibit the growth of released cells.

As expected, CAase treatment alone disrupted the biofilm structure and reduced the overall biomass for all *L. monocytogenes* biofilms (Figure 1, Figure 2 and Figure 3). Additionally, CAase alone increased the number of viable cells recovered from the treatment supernatants relative to PBS controls, which is consistent with enzymatic activity being limited to the disruption of the biofilm matrix and release of embedded cells (Figure 4 and Figure 5). This result demonstrated the need for additional “hurdles” to be included within the intervention strategy to prevent the subsequent spread of *L. monocytogenes* to the surrounding environment. For this study, both nisin and T110 were investigated due to their reported potent anti-listerial activities.

In this study, nisin treatment alone resulted in a significant reduction in the biofilm biomass for eight of the eleven *L. monocytogenes* strains tested. A previous study reported that a similar commercial nisin product (Nisaplin) had little or no effect against a mature *L. monocytogenes* F6854 biofilm [22], further suggesting that nisin efficacy in disrupting *L. monocytogenes* biofilms is strain-dependent. However, when nisin was used in combination with an additional antimicrobial, citric acid or cinnamaldehyde, a significant reduction in viable cell numbers was observed within the mature biofilms. In a separate study, a combination of nisin and sage extract was effective in preventing biofilm formation by *L. monocytogenes* ATCC 19115 [26]. These studies suggest that nisin might function optimally when used with other “hurdles” to control *L. monocytogenes* biofilm formation and persistence. When combined with CAase, nisin was observed to enhance biofilm dispersal for two *L. monocytogenes* strains, 10403S and BAA-3132, significantly (Figure 1). Additionally, nisin resulted in significantly lower numbers of viable cells within the supernatant when compared to CAase treatment alone (Figure 4). This suggests that nisin inhibited the growth or killed the cells released due to the CAase-induced degradation of the biofilm matrix.

When T110 was used alone, a slight reduction in biofilm biomass was observed for two strains, 19111 and 19113 (Figure 2), suggesting that T110 was not active against cells embedded within biofilms for the remaining nine strains. Although the concentration of T110 used in biofilm assays (1024 AU mL^−1^) was higher than the MIC determined for all eleven strains, it may not be sufficient to inactivate cells embedded within a mature biofilm. However, an increase in viable cells within supernatants was observed for five strains after treatment with T110 alone when compared to their respective controls (PBS) (Figure 5). This suggests that T110 may alter biofilm structure, resulting in the release of viable cells, and agrees with the apparent visual changes in biofilm structure observed microscopically (Figure 3). When T110 was combined with CAase, only two strains, 10403S and 19118, showed additional reduction in biomass when compared with CAase alone. This is similar to what was observed with nisin and could be the result of how efficacious CAase is at removing biofilms. The remaining biomass observed in Figure 1 and Figure 2 could be cells tightly attached to the well surface that cannot be dislodged without physical intervention. T110 appeared to suppress the growth of viable cells released from biofilms for six of the strains tested, including 19111, which was used in the initial study assessing the efficacy of CAase and T110 against *L. monocytogenes* biofilms [15]. Results from this study showed that 19111 had the lowest MIC for T110, but its MBC was determined to be 2048 AU mL^−1^, suggesting T110 is needed in higher concentrations to fully lyse *L. monocytogenes* cells.

The enzyme–bacteriocin combinations described here show practical promise for application in food manufacturing and sanitation systems. CAase and nisin are derived from food-safe microbial sources and are compatible with existing cleaning infrastructure, suggesting that integration into clean-in-place or spray-based sanitation processes may be feasible. While T110 also showed promise against some *L. monocytogenes* strains, results here confirm that more work is needed to increase its production by *S. thermophilus* or develop more concentrated working solutions. However, this work was conducted under controlled laboratory conditions using single-species biofilms and standardized surfaces. Future investigations should evaluate the performance of these treatments under industrially relevant conditions that include mixed-species biofilms, varied surface materials, and environmental challenges such as organic load and temperature fluctuation. Long-term studies are also needed to assess the impact of repeated enzyme and bacteriocin exposure on microbial communities and to identify molecular and structural factors contributing to strain-dependent variability. Together, these insights will be essential for refining enzyme–bacteriocin combinations as food-safe, environmentally friendly, biologically based strategies for controlling biofilms in food production environments.

## 5. Conclusions

This work expands previous single-strain investigations to a broader and genetically diverse panel of *L. monocytogenes* isolates. The results confirm that pairing an extracellular matrix–degrading enzyme with a bacteriocin can disrupt established biofilms and reduce the number of viable cells released. In this study, nisin was more effective than T110 at inhibiting the recovery of viable cells, but T110 could be studied further in combination with nisin to prevent the survival of nisin-resistant *L. monocytogenes*. Additionally, the biomass remaining after treatment with CAase and nisin suggests that this intervention strategy may function best as part of a hurdle technology with additional biofilm-degrading compounds. The findings in this study support continued development of enzyme–bacteriocin combinations as food-safe agents for targeted biofilm control. Future studies should investigate the molecular basis of strain-specific responses and evaluate treatment efficacy under processing-relevant conditions to optimize this dual-action strategy for practical application in food manufacturing environments.

## Figures and Tables

**Table 1:** *Listeria monocytogenes* strains used in this study.

Strain	Serotype	Isolation Source	Notes
10403S	1/2a	Human	Gift from Daniel Portnoy; Available: BEI Resources (NR-13223)
ATCC 19111	1/2a	Animal/Poultry	Purchased ATCC
ATCC 19113	3	Human	Purchased ATCC
ATCC 19117	4d	Animal/Sheep	Purchased ATCC
ATCC 19118	4e	Animal/Poultry	Purchased ATCC
BAA-3131	1/2a	Food production environment	Purchased ATCC (FDA)
BAA-3132	4a	Food&Beverage	Purchased ATCC (FDA)
BAA-3134	4b	Cheese	Purchased ATCC (FDA)
BAA-3135	1/2b	Ice Cream	Purchased ATCC (FDA)
EGDe	1/2a	Animal/Rabbit tissue	In-house collection [23]; Available: ATCC (BAA-679)
F2365	4b	Jalisco soft cheese	In-house collection [24]

ATCC: American Type Culture Collection (Manassas, VA, USA); BAA: Biological Assessment Assurance; FDA: Food and Drug Administration.

**Table 2:** Minimum inhibitory concentration (MIC) of nisin and T110 against *L. monocytogenes* strains.

*L. monocytogenes* Strain	Nisin (µg mL^−1^)	T110 (AU mL^−1^)
10403S	12.5	128
ATCC 19111	12.5	64
ATCC 19113	12.5	512
ATCC 19117	12.5	128
ATCC 19118	12.5	256
BAA-3131	25	256
BAA-3132	25	256
BAA-3134	25	256
BAA-3135	12.5	256
EGDe	12.5	256
F2365	12.5	512

## Data Availability

The original contributions presented in this study are included in the article. Further inquiries can be directed to the corresponding author.
